# Cognitive Process of Psoriasis and Its Comorbidities: From Epidemiology to Genetics

**DOI:** 10.3389/fgene.2021.735124

**Published:** 2021-11-26

**Authors:** Jing Gao, Xue Shen, Randy Ko, Cong Huang, Changbing Shen

**Affiliations:** ^1^ Department of Dermatology, the Second Affiliated Hospital, Anhui Medical University, Hefei, China; ^2^ Department of Dermatology, Chengdu Second People’s Hospital, Chengdu, China; ^3^ Department of Internal Medicine, University of New Mexico Health Sciences Center, Albuquerque, NM, United States; ^4^ Department of Dermatology, Peking University Shenzhen Hospital, Shenzhen, China; ^5^ Shenzhen Key Laboratory for Translational Medicine of Dermatology, Shenzhen Peking University–the Hong Kong University of Science and Technology Medical Center, Shenzhen, China

**Keywords:** psoriasis, comorbidity, epidemiology, genetics, cognitive

## Abstract

Psoriasis (PsO) is a chronic inflammatory skin disease that affects approximately 2% of the population all over the world. Comorbidities of PsO have increasingly garnered more interest in the past decades. Compared with the normal population, the incidences of comorbidities are higher among patients with PsO. In the last 20 years, researchers have focused on studying the genetic components of PsO, and genetic associations between PsO and its comorbidities were elucidated. This review provides an in-depth understanding and summarization of the connection between PsO and its comorbidities from the perspectives of epidemiology and genetics. Further understanding of PsO and its comorbidities will promote research on the pathogenesis, drug development, novel therapy methods, and personalized and precision treatment of PsO and its comorbidities.

## 1 Introduction

Psoriasis (PsO) is an immune-mediated chronic inflammatory skin disease, mainly characterized by erythema and scales on the skin with clear skin lesion boundaries (Griffiths et al., 2021). PsO must be differentiated from seborrheic dermatitis, pityriasis rosea, secondary syphilis, lichen planus, and chronic eczema. Currently, treatment methods and therapy effects of PsO are limited and unsatisfactory; although biologics have shown better efficacy, the prices are expensive and require long-term maintenance treatment (Bagel et al., 2021; Brownstone et al., 2021; Xie et al., 2021). PsO is prone to recurrent episodes, which brings difficulties and challenges in the management of PsO. In addition, the treatment of PsO is not only limited to skin manifestations but also present with comorbidities that already exist or occur in the course of PsO. These further increase the difficulty and complexity of treatment for PsO. Understanding and being cognizant of the comorbidities of PsO has shown to be important for its comprehensive diagnosis and treatment (Kanda, 2021). In recent years, numerous epidemiological and genetic studies have focused on the comorbidities of PsO, which has greatly expanded our understanding of PsO and its comorbidities.

## 2 Related Terminology

In clinics, we often use terminologies such as “comorbidity”, “complication”, “secondary disease”, and “concomitant disease” to describe the phenomenon that multiple diseases exist at the same time within a patient. However, the definition and scope of these terminologies are both overlapping and different. In some studies, these terminologies may be confused and misused. Therefore, it is necessary to master the definitions of these terminologies and use them correctly. Before clarifying and understanding the definition of these terminologies, it is necessary to grasp the definition of “primary disease”, which refers to the disease that appears first in the body with pathological changes, and it can be dominant or insidious (Liu, 2011). In some patients, when two or more diseases appear first at the same time, and there is no causal relationship between them, this may be described as “coordinate primary diseases”. The terminology “secondary disease” refers to a disease that occurs after the primary disease and has a causal relationship with it (Liu, 2011).

Until now, there is no completely accepted definition of the terminology of “comorbidity” and “complication”, and they are easily confused. Researchers may have different opinions on the definition of these two terminologies (Liu, 2011; Radner et al., 2014). “Comorbidity” is defined as a medical condition coexisting with the primary disease, which refers to the simultaneous or sequential occurrence of two or more diseases in the body. Additionally, a comorbidity may be a current condition or a past condition and may be related or not related to the primary disease. Lastly, “complication” refers to the occurrence of a disease that is related to the primary disease, and there is a causal relationship with the primary disease. Zuo and Tang (2013) described when there is a causal relationship between two diseases, it is more appropriate to use “complication”. Furthermore, “comorbidity” can be utilized if there is no causal relationship between the two diseases, or if the causal relationship is not easy to determine. This contributes to better understanding of the appropriate use of “comorbidity” instead of “complication”. According to the understanding by the authors of this review, the conception and scope of “comorbidity” is closer to the accompanying disease. Hence, “comorbidity” is used to describe coexisting diseases of PsO.

## 3 Epidemiological Characteristics of Psoriasis and its Comorbidities

As early as 1897, Kawazoe et al. (1897) reported an association between PsO and diabetes mellitus (DM), which is the first reported observed comorbidity in PsO. In 1961, Reed et al. described that PsO patients with psoriatic arthritis (PsA) had a higher proportion of deaths due to heart diseases Reed et al. (1961). McDonald and Calabresi (1978) found an increased prevalence of venous and arterial vascular diseases in PsO hospitalized patients. In recent decades, the comorbidities of PsO have increasingly gained more interest to clinicians and researchers. Additional epidemiological studies have shown that one or more kinds of comorbidities are accompanied with PsO patients. These mainly include autoimmunity, neurological, and cardiometabolic diseases which lead to an increase in mortality and severity of PsO. Although there have been a number of studies reporting the associations between PsO and its comorbidities, the underlying mechanism is complex. Additionally, treatment interventions and biases may confuse the relationships between PsO and its comorbidities. Genetic susceptibility and overlapping inflammatory pathways are speculated to be the potential biological connection of these associations (Sales and Torres, 2014).

At present, researchers around the world have carried out various forms of research studies to explore the epidemiological characteristics of PsO and its comorbidities. These include: a) Case report (Supplementary Table S1): these diseases are generally found during the clinical diagnosis and treatment, reported in the form of cases. Typically, the numbers of cases are small, such as alopecia areata (Khaled et al., 2012), T-cell leukemia (Koga et al., 2012), and acquired immune deficiency syndrome (AIDS) (Blanco González et al., 2000). For the comorbidities of PsO reported in the form of a case report, more clinical observation data is needed to further clarify the accompanying phenomena and correlation relationships in the future. b) Cross-sectional study: through the cross-sectional study, it can be found that a certain proportion of comorbidities occur in patients with PsO, or vice versa. This type of research indicates a concomitant phenomenon, which further indicates the clustering of comorbidities in PsO. c) Case-control study: in case-control studies, health controls are usually compared to the occurrence of comorbidities in PsO patients. The odds ratio (OR) of a certain comorbidity in the population of PsO patients can be calculated, or the OR of PsO in the population with a specific disease can be calculated. d) Cohort study: similar to the case-control study, with the normal population as the control group, the risk ratio (RR) of a certain comorbidity in the population of PsO patients can be calculated, or the RR of PsO in the population with a special disease can be calculated. e) Meta-analysis: a large number of studies related to PsO and its comorbidities (such as cross-sectional study, case-control study, cohort study, etc.) have been carried out and published. However, there are inconsistencies in the results of these studies; therefore, meta-analyses are performed and are helpful in providing more sufficient evidence to show the correlation between PsO and its comorbidities. f) Systematic review (Supplementary Table S1): researchers reviewed the accompanying phenomena of PsO and other diseases while summarizing the observed phenomena and research findings while putting forward hypotheses. In addition, the use of a combination of research methods has helped discover the epidemiological characteristics of PsO and its comorbidities, such as cross-sectional retrospective study and retrospective cohort study.

In this review, we summarized the epidemiological characteristics of PsO and its comorbidities, including skin diseases (Table 1), tumors or cancers (Supplementary Table S2), cardiovascular diseases (Supplementary Table S3), neuropsychiatric disorders (Supplementary Table S4), diseases of the digestive system (Supplementary Table S5), metabolic diseases (Supplementary Table S6), and some other diseases (Supplementary Table S7).

**TABLE 1:** Summarization of epidemiological studies between PsO and its comorbidities (skin diseases)^a^.

Comorbidities	Description	Rresearch type	Ref
Psoriatic arthritis	A total of 266 studies examining 976,408 patients with PsO were included. 19.7% PsO patients with PsA (95% CI: 18.5–20.9%)	Meta-analysis	Alinaghi et al. (2019)
Vitiligo	10 case-control/cross-sectional studies with a total of 120,866 PsO cases and 79,907 vitiligo cases. Higher incidence of PsO in vitiligo patients than healthy controls (total OR = 2.29, 95% CI: 1.56–3.37)	Meta-analysis	Yen and Chi (2019)
Contact dermatitis	The database included 887,765 adolescents, of whom 3,112 (0.35%) were diagnosed with PsO. Contact dermatitis was significantly associated with PsO (OR = 2.26, 95% CI: 1.43–3.55)	Cross-sectional study	Shreberk-Hassidim et al. (2019)
Atopic dermatitis	A retrospective chart review was performed, and data were recorded for patients with PsO (*n* = 1,390), atopic dermatitis (*n* = 912), and PsO plus atopic dermatitis (*n* = 30). The comorbid ratio of atopic dermatitis and PsO was 1.5%	Retrospective study	Barry et al. (2019)
Bullous pemphigoid	Four case-control studies with a total of 4,035 bullous pemphigoid patients and 19,215 healthy controls for inclusion in the meta-analysis. Bullous pemphigoid with a higher incidence of PsO (OR = 2.5, 95% CI: 1.4–4.6) compared with healthy controls	Meta-analysis	Phan et al. (2019)
Systemic lupus erythematosus	42 individuals were diagnosed with systemic lupus erythematosus in 9,420 PsO patients	Retrospective study	Zalla and Muller (1996)
	150 of 20,630 SLE hospitalizations have co-existing PsO	Retrospective study	Ojemolon et al. (2020)
Pemphigus	12 eligible studies comprising 12,238 patients with pemphigus were included in the quantitative synthesis. 2.4% PsO patients have pemphigus (95% CI: 1.0–4.4)	Meta-analysis	Kridin et al. (2019)
	4 studies with 11,183 pemphigus cases and 87,051,171 healthy controls were pooled in the meta-analysis. Pemphigus was more common in patients with PsO than in healthy controls (OR = 2.64, 95% CI: 1.24–5.59)	Meta-analysis	Phan et al. (2020)
Non-melanoma skin cancer	A total of 16 cohort studies involving 16,023,503 participants met the inclusion criteria and were included in the final analysis. PsO patients have a higher risk of non-melanoma skin cancer than controls (RR = 1.72, 95% CI: 1.46–2.02)	Meta-analysis	Wang et al. (2020)

a There is more than one study about PsO and its comorbidities that has been conducted. Here, we listed the study with a bigger sample size and refer to the evidence level.

Note: OR: odds ratio; aOR: adjusted odds ratio; CI: confidence interval.

## 4 Research Progress in Genetics of Psoriasis and Its Comorbidities

Genetic studies [especially genome-wide association study (GWAS)] have identified many susceptibility genes for skin diseases (Zhang, 2012; Sun et al., 2016), thereby expanding the understanding of the genetic pathogenesis of these diseases. Interestingly, studies have found that there are some common genes/loci between PsO and its comorbidities. These comorbidities mainly include PsA, atopic dermatitis (AD), vitiligo, systemic lupus erythematosus (SLE), alopecia areata (AA), rheumatoid arthritis (RA), inflammatory bowel disease (IBD), cardiovascular diseases (CVD), DM, metabolic syndrome (MSy), schizophrenia (SCZ), HIV infection, and chronic periodontitis. This article reviews the common susceptibility genes and genetic pathogenesis between PsO and its comorbidities.

### 4.1 Common or Differential Susceptibility Genes

#### 4.1.1 Psoriatic Arthritis

20-30% of PsO may progress to PsA, and epidemiological studies have shown that PsA has a connection to heritability (O’Rielly et al., 2019). Early researchers focused on PsA and PsO susceptibility loci and compared the susceptibility regions related to these two diseases. It was found that the susceptibility regions of these two diseases may include an overlap in the range of 100 kb between HLA-C and OTF3 (Martinez-Borra et al., 2003). Sakkas et al. (1990) found that HLA-DR7a exists in 38.8% of PsA patients, 41.5% of PsO patients, and 8% of healthy individuals, indicating that HLA-DR7a may be an important susceptibility factor for PsA and PsO. Loft et al. (2018) found that TNF- [single nucleotide polymorphism (SNP) rs361525] was significantly associated with PsO, cutaneous PsO (PsC; PsO patients with a history of at least 10 years and without the manifestation of arthritis), and PsA. A meta-analysis of the genotypes and/or allele frequencies of TNF-α promoters in PsO vulgaris (PsV), PsA, and healthy controls, which showed that the variant genotypes and alleles of TNF-α-308A/G are found in PsV and PsA patient groups and demonstrate a protective effect (Zhu et al., 2013a). However, the variant genotypes and alleles of TNF-α-238A/G and -857T/C have the effect of increasing the risk of PsV and PsA (Zhu et al., 2013a).

To explore whether the variation in PsV-related locus is also related to PsA, Zhang et al. (2017) conducted a candidate loci association study in Chinese PsA patients. This was carried out by performing genetic studies on 50 SNPs that have been reported to be associated with PsO. The results showed that the SNPs in 5q33.3, 1p36, and 1q21.3 are strongly associated with PsA, and IL12B, RUNX3, and LCE are candidate genes for these regions. Yang et al. (2013) genotyped 20 SNPs in the Chinese population cohort (PsO, PsA, and healthy controls), and results showed that TNIP1 (rs17728338), IL28RA (rs4649203), IL12B (rs2082412), ERAP1 (rs27524), PTTG1 (rs2431697), and GJB2 (rs3751385) were correlated with PsA. Furthermore, SNP IL28RA (rs4649203), TNIP1 (rs17728338), and ERAP1 (rs27524) were significantly correlated with PsC. There was no statistical difference in the allele frequency of SNPs in IL12B and ZNF816A between PsA and PsO. These results support the hypothesis that PsA and PsO have similar genetic etiology and that the genetic component of PsA is higher than that of PsO.

IL23R and IL12B are susceptibility genes for a variety of autoimmune and inflammatory diseases (Liu et al., 2015). Liu et al. (2008) performed genotyping of PsO (including PsA) and compared the results with those of the Nordic healthy controls, whereas independent verifications were conducted in two populations (PsO and healthy controls from United States, and PsA and healthy controls from United Kingdom). The previously reported associations with IL23R (rs11209026) and IL12B (rs6887695) polymorphisms were verified in the PsO and PsA cohorts. Zhu et al. (Zhu et al., 2012; Zhu et al., 2013b) utilized a meta-analysis method to explore the relationships of IL23R polymorphisms [rs11209026 (Q381R), rs7530511 (L310P), and rs2201841] and IL12B polymorphisms (rs6887695 and rs3212227) with PsO or PsA susceptibility, with the results proving that there is a significant correlation of IL23R and IL12B polymorphisms with PsO or PsA.

In the British population, 20 SNPs were genotyped in patients with chronic plaque PsO [including subgroup data of diagnosed PsA and early onset (age of onset <40 years old) PsO]. Comparing the allele frequency of PsO patients with the Welcome Trust Case Control Consortium (WTCCC) controls, it was found that there is a significant association between the missense variant rs4986790 (Asp229Gly) of TLR4 and chronic plaque PsO, which was also notable in those with PsA and early onset PsO (Smith et al., 2016). GWAS was also carried out in German PsV and healthy controls, whereas 147 most significant SNPs were verified in 3 independent groups (Ellinghaus et al., 2010). A stratified analysis showed that TRAF3IP2 is effective against PsA, with a common susceptibility to PsV (Ellinghaus et al., 2010). In another German cohort, sequencing of all exons of TRAF3IP2 in PsA patients revealed a further coding variant (rs33980500, p.Asp10Asn), genotyping showed an even stronger association with PsA as well as association to PsV (Hüffmeier et al., 2010).

Bowes et al. (2017) performed genotyping of human leukocyte antigen (HLA) which showed that HLA-C*06:02 is not related to PsA and that the relationship is different between PsA or PsC and HLA-B. A family-based association study was used to confirm whether some specific HLA alleles of PsO may cause PsA, and the results showed that HLA-C*12, HLA-B*38, HLA-B*39, and HLA-B*27 alleles are more frequently found in PsA than PsO, indicating that these alleles are potential specific genetic markers for PsA in PsO patients (Eder et al., 2012). Liao et al. (2008) evaluated the genetic role of HLA in patients with PsO and PsA in a Chinese population, with the frequency of HLA-B*27 and HLA-Cw*12 being higher in PsA patients than PsO patients, while the frequency of HLA-DR*07 is higher in PsO patients. In the same study, HLA-B*27 is significantly associated with axial joint involvement and uveitis in PsA patients; therefore, this indicates that PsO patients with HLA-B*27 and/or -Cw*12 may have a higher risk of PsA. Ho et al. (2008) studied the susceptibility of HLA-Cw*06 and HLA-DRB1 to PsA and found that HLA-Cw*06 is correlated to PsA with type I PsO (age of onset <40 years old), but no correlation with PsA with type II PsO (age of onset ≥40 years old) was found. HLA-DRB1*07, which is in linkage disequilibrium (LD) with HLA-Cw*06, is also associated with PsA with type I PsO, and HLA-Cw*06 and HLA-DRB1*07 are associated with PsA with type I PsO, indicating that the main association is related to the onset age of PsO. PsA with type I and type II PsO have different genetic backgrounds. Therefore, it is necessary to use the onset age of PsO as a stratification factor to analyze the genetic susceptibility of PsA in future studies.

The association between the three common variants [NFKB1 (rs230526), NFKBIA (rs7152376), and NFKBIZ (rs3217713)] and the risk of PsO progression were explored (Coto-Segura et al., 2019). NFKBIA rs7152376*C was significantly higher in PsA than controls, and the difference between PsC patients had a stronger association, indicating that NFKBIA has a significant effect on the risk of PsO progressing to PsA (Coto-Segura et al., 2019). Additionally, there were twelve SNPs that were genotyped in PsA, PsC, and healthy controls in the Chinese population. A variant (rs12883343) in NFKBIA was shown to be significantly different from PsA and PsC, which indicates that the role of NFKBIA in the pathogenesis of PsA and PsC is different (Zhao et al., 2018). In order to distinguish the differences in genetic risk factors of PsA and PsC, Stuart et al. (2015) conducted a GWAS of PsA and GWAS meta-analysis (including PsV, PsA, and PsC) of three other European populations. It was found that ten regions were related to PsA and eleven regions were related to PsC with statistical significance at the whole genome level. Among the identified risk variants, three loci (rs12189871 near HLA-C; rs490874 near TNFRSF9; and rs10888503 near LCE3A) are more closely related to PsC. Furthermore, two loci (rs12044149 near IL23R; rs9321623 near TNFAIP3) are more closely related to PsA. PsA-specific mutations are independent of previously identified PsO mutations near IL23R and TNFAIP3. These research findings provide insights into the similarities and differences between PsC and PsA.

A study on the relationship between smoking and IL13 polymorphisms with PsA and PsO (Eder et al., 2011) demonstrated that rs20541*G and rs848*C alleles were associated with PsA when compared to healthy controls. Additionally, an association between these alleles and PsC were shown to have borderline significance. Compared with PsC, the frequency of the two main alleles was increased in PsA. The combination of non-smoking and genotype rs1800925*CC was associated with an increased susceptibility to PsA. Among smokers, rs1800925*CC is not related to PsA when compared to PsC. Bowes et al. (2015) selected fifteen SNPs for verification in independent PsA case-control groups and performed meta-analysis with immunochip data. Comparing the new PsA susceptibility locus with data from two large PsO studies (WTCCC2 and immunochip), it was found that rs2476601 (PTPN22) is significantly associated with PsA, but there is no evidence of an association in PsO. This demonstrates significant differences in the correlation of PsA and PsO.

Soto-Sanchez et al. (2010) analyzed the CCR5-32bp deletion (DeltaCCR5), CCR5 promoter (rs1799988), and CCR2-I64V (rs1799864) polymorphisms in PsO patients and healthy controls from Spain. There were no differences in allele and genotype frequencies between patients and healthy controls. However, compared with PsO patients without arthritis, the frequency of CCR2-64I carriers in PsO patients with arthritis was significantly higher, indicating that genetic mutations in the CCR2/CCR5 gene do not increase the risk of PsO, but CCR2 polymorphism may influence the risk of arthritis in PsO. Mossner et al. (2005) analyzed the TNFA-238 and TNFA-308 genotypes in PsV patients, palmoplantar pustulosis PsO (PPP) patients, and healthy controls. The results showed that carrying the TNFA-238*A genotype is related to PsV in the white population and carrying TNFA-308*A may demonstrate a protective effect. Compared with healthy controls, although the carrying of TNFA-308*A in PsA is reduced, it may be a sign of more severe joint involvement in arthritis patients. These findings support the difference between TNFA polymorphisms in PsV and PsA. Hüffmeier et al. (2009) explored whether there are differences in the risks of PsO skin and joint performance-related variants, specifically in four variants of the IL12B and IL23R genes in PsV patients, PsA patients, and healthy controls. For PsA and PsV, the strongest association existed in two IL12B SNPs (rs6887695 and rs3212227), and the corresponding haplotypes were detected. Specifically, for IL23R, only rs11209026 showed an association. No differences were observed after stratification of the risk alleles of PsO susceptibility locus 1 (PSORS1), indicating that these variants are not specific risk factors for arthritis and are usually related to the susceptibility of PsO.

Frequently, clinicians and patients are concerned with the likelihood of PsO patients progressing to PsA. Bowes et al. (2011) genotyped two SNPs (rs20541 and rs1800925) of IL-13 in patients with PsA and PsV. The results showed that both SNPs were found to be highly associated with susceptibility to PsA, but neither SNP was significantly associated with susceptibility to PsV. This study shows that the role of IL-13 risk locus is specific to PsA, thus highlighting the differences in the key biological pathways of PsA and PsV. This may provide the possibility for future screening of PsV patients to identify patients at risk of developing PsA. Patrick et al. (2018) found 200 genetic markers to distinguish PsA after utilizing genotype data of >7,000 PsA and PsC patients in six cohorts. Among the top 5% of PsA prediction, > 90% precision with 100% specificity and 16% recall for predicting PsA was achieved among psoriatic patients, using conditional inference forest or shrinkage discriminant analysis. These research findings are important for distinguishing different types of psoriatic diseases, which possess the potential to be translated into clinical applications in the future.

#### 4.1.2 Atopic Dermatitis

AD and PsO are two common immune-mediated inflammatory skin diseases. As early as 2001, Cookson et al. (2001) carried out a genome screen of AD and identified a correlation of AD with the chromosomes 1q21, 17q25, and 20p. Interestingly, these regions are very close to the known PsO locus, as is the previously identified AD locus 3q21, indicating that these regions have an effect on skin inflammation and immunity. Filaggrin (FLG) is a very significant susceptibility gene for AD, but the two loss-of-function mutations in FLG-R501X and -2282del4 have no correlation with the susceptibility of PsO and AD patients in the Italian population (Giardina et al., 2008). Shi et al. (2016) assessed the relationship between rs4722404 (CARD11) and the risk of PsV in the southern Chinese Han population, but no association was found between rs4722404 and the risk of PsV. Hierarchical analysis based on the age of onset, family history, PsO area and severity index (PASI) score showed that there is a significant correlation between the C allele and CC + CT genotype of rs4722404 and an increased risk of early-onset PsV compared to that of late-onset PsV (onset age ≥40 years old).

Regarding whether there are some common susceptibility genes between AD and PsO, researchers from Japan have conducted a lot of research to clarify the pleiotropy of some genes in these two diseases. a) Tamari et al. (2014) explored the correlation between 41 SNPs out of 36 susceptibility gene loci of PsV and AD patients in the Japanese population. They found a marginal association between AD and two PsO-susceptible SNPs: IL13 (rs1295685, opposite direction effect) and ZMIZ1 (rs1250546, same direction effect). b) Kato et al. (2011) assessed whether CYSLTR2-1220 A⁄C is a susceptibility genetic factor for AD or PsV in the Japanese population and found that CYSLTR2-1220 A⁄C was not correlated with AD or PsV patients. This indicates that CYSLTR2-1220 A⁄C is not a major role at least in the development of AD or PsV in the Japanese population. c) IL-17F is a member of the IL-17 family. The IL-17F gene is located on 6p12, and encodes IL-17A and IL-17F. Studies have shown that IL-17F SNP 7488 T/C may not be related to PsV or AD in the Japanese population. Further research on other SNPs of the IL-17F gene in the Japanese population and SNP 7488 T/C in other populations would be beneficial in understanding the pathogenesis of PsV and AD (Shibata et al., 2009). d) The IL4R polymorphisms have been previously determined to be related to AD: 3112C > T, 1803T > C, 327C > A, 326A > C, and 186G > A. Fukai et al. (2004) explored whether these polymorphisms are related to PsO in the Japanese population and found that IL4R 326A > C and 186G > A variants are significantly associated with PsO. Interestingly, these allelic associations are opposite to the correlation effects found in AD patients in the Japanese population. e) IL-12 is considered to play an important role in inducing the Th1-type cytokine profile, and AD and PsV are considered to be Th2-mediated and Th1-mediated diseases, respectively. Tsunemi et al. (2002) explored whether the SNP of IL12B (1188A/C) is related to the susceptibility to AD or PsV in the Japanese population. Compared with the controls (50.5%), the A allele of AD patients decreased (40.9%), while the A allele of the PsV patients increased (60.1%). This indicates that IL12B (1188A/C) may affect the balance of Th1/Th2 of AD and susceptibility to PsV.

Tsoi et al. (2019) conducted a large-scale transcriptomics study, in addition to comparing with previous AD transcriptomics studies and comparing with PsO data in the same cohort. Studies have found that there is a surprising correlation between PsO dysregulated genes and AD, with 81% of AD dysregulated genes being shared with PsO. However, with regard to disease-specific molecular and cellular characteristics, AD skin shows a dominance in the IL-13 pathways, but IL-4 expression is almost undetectable. The proportion of long non-coding RNAs with disease heterogeneity and disorder is greater in AD. This study proved the shared inflammatory components and specific inconsistent cytokine characteristics between PsO and AD through RNA sequencing. Baurecht et al. (2015) used the GWAS and immunoChip datasets and developed a meta-analysis method to compound the epidermal differentiation complex (1q21.3), Th2 locus control region (5q31.1), and major histocompatibility (MHC). In the body (6p21-22), the risk alleles of the shared locus and the independent disease-specific locus are determined. It is further confirmed that the previously unreported pleiotropic alleles (PRKRA and ANXA6/TNIP1) have opposite effects on the risks of AD and PsO. This study shows that AD and PsO have unique genetic pathogenesis and have opposite effects in the shared pathways affecting epidermal differentiation and immune response.

In addition, childhood AD GWAS shows that there is a genetic link between AD and PsO. Weidinger et al. (2013) analyzed the SNPs related to PsO previously discovered by the GWAS. About two-thirds of the related variants showed opposite risk characteristics for AD and PsO, and the remaining one-third showed the same allele. Of these genes, the most significant association with AD is the SNP rs1295685 at the IL13 locus, which has the opposite effect of AD and PsO at the genome-wide level. These patterns may reflect the common and different characteristics of the two common skin diseases caused by genetic factors, and the relative risk tendency is not highly consistent with the frequency of the two diseases.

#### 4.1.3 Vitiligo

Vitiligo is an autoimmune disease with a strong genetic component, characterized by areas of depigmented skin (Shen et al., 2016). The PSOR1 locus (6q21) is closely related to PsO, but due to the strong LD in the MHC region, it is difficult to determine if it is an independent association. Zhu et al. (2011) performed a stepwise regression analysis of more than 3,000 SNPs in the MHC region and obtained four regression models in the Chinese Han PsO patients and healthy controls, among which the SNPs in HLA-C/HLA-B (rs9468925) and HLA-DQA2 (rs2858881) are verified in all models. This indicates that multiple loci, other than the PSOR1 locus, are related to PsO. More importantly, this study found that rs9468925 in HLA-C/HLA-B is related to both PsO and vitiligo, providing important evidence for the common genetic loci of these two skin diseases. This lays a foundation for the elucidation of the molecular mechanisms of skin diseases.

The X-linked mutation rs2294020 is located on exon 7 of the CCDC22 gene. The protein encoded by the CCDC22 gene plays a role in regulating NF-κB and is the main regulator of immune response. The rs2294020 polymorphism is likely to be a general susceptibility factor for autoimmune diseases. D’Amico et al. (2017) found that rs2294020 has a significant correlation with SLE, vitiligo, and PsO in European populations, indicating that rs2294020 is associated with several autoimmune-related diseases (especially involving in skin).

Cytotoxic T lymphocyte antigen 4 (CTLA-4) is a key negative regulator of T-cell activation and proliferation. Studies have evaluated the association between CTLA-4 + 49A/G polymorphism and PsO or vitiligo, but the results are inconsistent. Liang et al. (2015) conducted a meta-analysis of 6 PsO and 8 vitiligo studies to explore the relationship between the CTLA-4 + 49A/G polymorphism and the susceptibility to PsO and vitiligo. Overall, no significant correlation was found between CTLA-4 + 49A/G and PsO. The overall analysis and subgroup analysis based on race, genotype frequency, and heterogeneity failed to prove the association between CTLA-4 + 49A/G polymorphism and vitiligo.

#### 4.1.4 Systemic Lupus Erythematosus

SLE is an autoimmune disorder associated with nearly 100 susceptibility loci (Yin et al., 2020). Li et al. (2013) studied 20 SNPs in seventeen previously reported PsO susceptibility loci and 34 SNPs in 24 previously reported SLE susceptibility loci in the GWAS dataset of PsO and SLE. Among these SNPs, two SNPs (rs8016947 and rs4649203) were associated with these two diseases and were selected for further verification in independent PsO and SLE samples. The results showed that NFKBIA (rs8016947) and IL28RA (rs4649203) have a significant level of correlation with PsO and SLE, indicating that NFKBIA and IL28RA are common susceptibility loci for PsO and SLE in the Chinese Han population. Further studies on the functions of these two genes and their roles in pathogenesis of PsO and SLE are needed in the future.

#### 4.1.5 Alopecia Areata

AA is a chronic autoimmune disease characterized by patchy non-scarring hair loss (Zheng and Tosti, 2021). PsO and AA are two Th1 cell–mediated inflammatory skin diseases, and there are cases of co-occurrence of PsO and AA being reported (Beaziz et al., 2021). Is there any intrinsic connection between these two diseases? Martinez-Mir et al. (2007) performed whole-genome scans on AA patients from the United States and Israel and healthy individuals and performed analysis by using several different statistical methods. This revealed that there are at least four susceptibility loci on chromosomes 6, 10, 16, and 18 and confirmed the previous correlation between the HLA locus and AA. It is worth noting that the main loci on chromosomes 16 and 18 are consistent with the reported PsO loci, indicating that these regions may contain genes related to many different skin and hair diseases.

#### 4.1.6 Rheumatoid Arthritis

Both RA and PsO are common autoimmune diseases. However, there are few research reports discussing the common genetic risk factors of RA and PsO, except for the HLA genes (Li and Begovich, 2009). In a British population cohort, Ali et al. (2013) verified eighteen SNPs previously reported to be associated with RA in patients with early-onset PsO and controls and found that early-onset PsO is associated with the REL (rs13031237). However, the minor allele of rs13031237 has the opposite effect on the susceptibility to PsO and RA. PSORS1C1/CDSN is the susceptibility gene of PsO. Sun et al. (2013) investigated whether PSORS1C1/CDSN was involved in RA and found that the TagSNPs rs3130983, rs3778638, and rs4959053 in the PSORS1C1/CDSN locus were shown to predict susceptibility to RA in two independent RA cohorts. Additionally, there are significant differences in the genotype frequencies between RA and healthy controls, with rs3778638 allele frequencies and genotype frequency being significantly related to RA. Western blot showed that the expression of PSORS1C1 in the RA synovial tissue was significantly increased, and ELISA detected higher levels of PSORS1C1 and CDSN in blood of RA patients. These findings indicate that the PsO susceptibility gene PSORS1C1 may play an important role in the development of RA.

#### 4.1.7 Inflammatory Bowel Disease

IBD includes Crohn’s disease (CD) and ulcerative colitis (UC). In terms of genetic research, some genetic associations between PsO and CD have been discovered, while there are relatively few studies on the association between PsO and UC. Previous studies have confirmed that IL23R is associated with IBD and IL23R is also associated with PsO, indicating that this gene may be an important candidate gene for many chronic inflammatory diseases. Einarsdottir et al. (2009) explored the association between Swedish IBD patients and single nucleotide variants in the IL23R gene, and also studied the same genetic variants in Finnish PsO patients. This study verified the association between IL23R and IBD in Swedish patients, and an association between IL23R and PsO was found in the Finnish population.

Wolf et al. (2008) explored the association between 15 CD-related loci and PsO in a data set of PsO cases and healthy controls. It was found that the loci are mapped to the chromosomes 1q24 (rs12035082), 6p22 (rs6908425), and 21q22 (rs2836754). It is worth noting that the marker showing the strongest phenotypic effect (rs6908425) is mapped to CDKAL1, which is also associated with type 2 diabetes mellitus (T2DM) (Dehwah et al., 2010). These results confirm that the CDKAL1 gene has a pleiotropic role in the pathogenesis of immune-mediated diseases. Quaranta et al. (2009) found that CDKAL1 SNP rs6908425 was significantly correlated with PsO by analyzing the PsO dataset. The association with PsO and CD was completely independent of the association with T2DM. Gene expression studies showed that there is no CDKAL1 transcript in skin keratinocytes, but CDKAL1 is expressed in large quantities in immune cells. CDKAL1 expression is especially present in CD4^+^ and CD19^+^ lymphocytes. When immune cells are activated by proliferation signals, CDKAL1 expression is significantly downregulated. These findings indicate that the CDKAL1 locus has allelic heterogeneity, and CDKAL1 alleles may endow clinical susceptibility to different diseases through different effects on disease-specific cell types.

Although epidemiology, pathology, and therapeutics have shown that PsO and CD have a certain connection, little is known about their common genetic factors. Ellinghaus et al. (2012) conducted a meta-analysis of 5 published GWASs. There are 20 loci that showed the strongest evidence for the common association with these two diseases, and it was verified in PsO, CD, and controls. This study identified 7 PsO and CD-shared susceptibility loci outside the HLA region (9p24 near JAK2, 10q22 near ZMIZ1, 11q13 near PRDX5, 17q21 near STATS3, 17q21 near SOCS1, 19p13 near YDJC, and 22q11 near FUT2), and four reported PsO and CD risk loci (IL23R, IL12B, REL, and TYK2) were verified. These results show the effectiveness of the joint analysis of clinically independent immune-mediated diseases and expanded the map of shared genetic risk loci.

Li et al. (2008) used genotyping of the marker SNPs in the 725 kb region defined by IL-3 and IL-4 to assess whether other variants in the 5q31 region are causally related to these SNPs or have independent contributions to the risk of PsO. Ninety SNPs were tested in one case-control sample set and nine significant markers were then tested in two other sample sets. All nine SNPs were found to be significant in a meta-analysis of the combined sample sets. The analysis results showed that the intergenic SNP rs1800925 is located upstream of IL-13, which can explain all the significant associations observed in SLC22A4 except for one SNP, rs11568506. Haplotype analysis of these two SNPs (rs1800925 and rs11568506) showed a significant increase in two common haplotypes (rs11568506-rs1800925: GC, GT). Several 5q31 region SNPs closely related to CD in the WTCCC study were not significant in PsO. These results identify the most significant risk variants of PsO in the 5q31 region and indicate that different 5q31 variants can cause CD and PsO risks.

The C insertion polymorphism (3020insC) in the NOD2 gene is a rare mutation associated with CD (Ogura et al., 2001). In addition, a susceptibility locus for PsO has been identified on the 16q region, which overlaps with the identified susceptibility locus for CD (Hugot et al., 1996). Young et al. (2003) proposed that NOD2 may be a candidate susceptibility gene for PsO and genotyped 3020insC of PsO to test this hypothesis. However, no statistically significant difference was observed between PsV, PPP, globular PsO, and healthy controls. In both patients and controls, no mutation homozygotes were observed. This specific insertion mutation in the NOD2 gene does not seem to contribute to the genetic susceptibility of PsV, PPP, or globular PsO. However, there are other mutations in the NOD2 gene that may play a role in PsO.

#### 4.1.8 Cardiovascular Diseases

The reciprocal phenomenon of CVD and PsO is more common in clinics, and a large number of literature has proven that these two kinds of diseases increase the risk of each other. However, the results of genetic studies indicate that there are some differences in genetic susceptibility.

##### 4.1.8.1 Coronary Artery Disease

To detect the potential genetic overlap among CAD and PsO patients, Koch et al. (2015) used GWAS datasets from > 22,000 cases of coronary artery disease (CAD) and > 4,000 cases of PsO, to perform genotyping studies on cardiometabolic risk loci in PsO cases and healthy controls, in order to detect the potential genetic overlap among CAD and PsO patients. The analysis results showed that, except for a weak signal from the MHC region, no evidence showed that there is a genetic risk locus between PsO and CAD. The results of this study indicate that the genetic structure of PsO and CAD are quite different.

##### 4.1.8.2 Coronary Artery Calcification

Torres et al. (2016) evaluated the effects of TNF-α polymorphisms [rs361525 (238G/A), rs1800629 (308G/A), and rs1799964 (1031T/C)] on coronary artery calcification (CAC) in patients with PsO. The results showed that these three TNF-α gene polymorphisms were not related to CAC. Therefore, it is necessary to further search for potential genes that may affect the development of CAC in PsO patients.

##### 4.1.8.3 Coronary Heart Disease

The findings from Hadi et al. show that although PsO may be a risk factor for CHD, psoriatic patients have a less severe CHD than the general population (Hadi et al., 2021). Gupta et al. (2013) used the GWAS catalog data to explore the share susceptibility loci between PsO and coronary heart disease (CHD). Interestingly, the genetic control of PsO is almost completely independent of CHD. With GWAS analysis, PsO susceptibility genes showed close clustering, while genes conferring susceptibility to CHD were interlinked separately. Based on these findings, researchers hypothesized that the coexistence of PsO and CHD observed clinically may be caused by common environmental factors (such as diet), and diet is considered a common risk factor.

##### 4.1.8.4 Hypertension

Ogretmen et al. (2014) explored the association between the eNOS (endothelial nitric oxide synthase) Glu298Asp polymorphism and the risk of hypertension in PsO patients in the Turkish population. Compared with PsO patients with normal blood pressure, the T allele frequency of eNOS Glu298Asp in PsO patients with hypertension is significantly different. This indicates that there is a correlation in the relationship between eNOS Glu298Asp polymorphism and hypertension in PsO patients in the Turkish population.

#### 4.1.9 Diabetes Mellitus

Epidemiological, immunological, and genetic studies have indicated that there are some biological links between PsO and DM (Shapiro et al., 2007; Azfar and Gelfand, 2008; Deng et al., 2016; Nekoua et al., 2016). In order to determine the shared susceptibility loci between these two diseases and explore the common pathogenesis, Wang et al. (2017) genotyped 89 DM susceptibility loci at 1p13 in PsO patients and healthy controls in the Chinese population. Three important associated signals were found in rs6679677 of 1p13.2, rs16861329 of 3q27.3, and rs849135 of 7p15.1, suggesting that *PTPN22, ST6GAL1, and JAZF1* are susceptibility genes for PsO in the Chinese population. This study shows that there may be some shared susceptibility genes between PsO and DM.

Eiris et al. (2014) analyzed the effects of SNPs (IL12B rs6887695 and rs3212227, IL23R rs2201841 and rs11209026, and IL23A rs2066808) associated with PsO on the main phenotypes and metabolic/cardiovascular characteristics of PsO patients in the northern Spanish population. It was found that there was an association between the IL23R rs11209026-GG genotype and more severe PsO, and there were significant associations between the three SNP genotypes (IL12B rs6887695-CC, IL12B rs3212227-CC, and IL23R rs2201841-GG) and T2DM. This study showed that in the population of northern Spain, genetic variants of IL12B, IL23R, and IL23A not only affect the risk of PsO but are also related to the severity of PsO and T2DM.

#### 4.1.10 Metabolic Syndrome

Abdel et al. (Abdel Hay and Rashed, 2011) studied the functional polymorphism of the LEP (G-2548A) in the genetic susceptibility of PsO and explored the factors affecting the plasma leptin level in PsO. Additionally, this group performed a stratified analysis based on the presence or absence of MSy. The study found that GG, GA and AA frequencies were 61.5%, 23.1% and 15.4% in PsO patients with MSy, respectively; while the distribution of genotypes was 29.4%, 50% and 20.6% in PsO patients without MSy, respectively. There is a statisticallly significant difference between these two groups. The plasma leptin level of the patients was significantly higher than that of the controls, and among the different LEP genotypes of the patients, the LEP G-2548A gene polymorphism may be a predictor of an increased plasma leptin level and increased risk of PsO.

#### 4.1.11 Schizophrenia

Pouget et al. (2019) observed the genetic correlation between PsO and SCZ through the analysis of genome-wide summary data. The degree and direction of the correlation are consistent with the phenotypic correlation predicted based on epidemiological data, indicating that PsO and SCZ share genetic risk factors. This may assist in explaining the epidemiological association that exists between PsO and SCZ. Yin et al. (2017) conducted PsO and SCZ whole blood expression quantitative trait loci (eQTL) analysis and observed a significant pleiotropic effect between PsO and SCZ. GWAS data analysis of PsO and SCZ shows the abundance of eQTL in whole blood, and reveals that the common variants of these two diseases are more likely to confer eQTL effects. In another study, genome-wide marker genotype datasets were used to explore the potential shared genetic susceptibilities between SCZ and PsO (Yin et al., 2016). This was performed by analyzing the genetic datasets of PsO, SCZ, and controls by verifying the results in an independent cohort. The results show that a large part of the risk of SCZ and PsO can be attributed to common variants, and the agreement between traits is estimated to be 21%. Five variants were identified in the HLA region, and they are most likely to be related to the two diseases with a significant risk effect. This study was the first that found that there is a common genetic etiology evidence between PsO and SCZ.

#### 4.1.12 Human Immunodeficiency Virus Infection

Does the enrichment of genetic variants that exist in PsO patients limit the ability of HIV-1 to replicate after infection? Chen et al. (2012a) analyzed the HLA-I and HLA-II alleles of PsO patients and controls and found that PsO patients have a greater possibility of controlling the genetic mutations of HIV-1 disease protection. This may suggest that enrichment of genetic variants that exist in PsO patients can limit the ability of HIV-1 to replicate after infection. Among them, it includes several HLA-I alleles related to HIV-1 control, with amino acid residues at positions 67, 70, and 97 of HLA-B that mediate HIV-1 peptide binding. Additionally, the deletion polymorphism rs67384697 is related to the high expression of HLA-C. Furthermore, it was also found that the compound genotypes KIR3DS1 and HLA-B Bw4-80I, which encode the natural killer cell activation receptor and its putative ligand, respectively, greatly increased the susceptibility of PsO. The compound genotype is also related to the delay in the progress of AIDS. The above-mentioned results indicate that genetic variation that contributes to antiviral immunity may promote the development of PsO.

Nititham et al. (2017) explored whether PsO-related SNPs affect the host control of HIV-1 infection in the three HIV-positive cohorts: a) HIV-1 controllers and non-controllers in the Study of the Consequences of the Protease Inhibitor Era (SCOPE) cohort study, b) Individuals with primary HIV infection in the options cohort, and c) HIV-positive injection drug users from the Urban Health Study (UHS). The results showed that two MHC variants (rs9264942 and rs3021366) of PsO were closely related to the status of the HIV-1 controller and viral load. Additionally it was determined that another variant (rs9368699) was strongly correlated with the viral load. Many genetic variants other than the MHC region (SOX5, TLR9, SDC4, PROX1, IL12B, TLR4, MBL-2, TYK2, and IFIH1) showed general significance. Overall, PsO variants within the MHC region have a strong influence on the control of HIV-1, while variants outside of the MHC region require further research.

#### 4.1.13 Chronic Periodontitis

The IFIH1 gene is a key gene that connects environmental and genetic factors in the pathogenesis of immune-related diseases, while playing a role in a variety of immune-related diseases. IFIH1 provides molecular links between genetic susceptibility, viral infections, and immune-related diseases. Chen et al. (2012b) explored whether IFIH1 gene has an effect on PsO and chronic periodontitis, and confirmed whether there is a common molecular mechanism between these diseases. Two common variants (rs1990760 and rs3747517) in IFIH1 were genotyped in PsO patients, chronic periodontitis patients, and the shared controls. The results showed that the allele distribution of these two SNPs in the Chinese population is drastically different from that in the European population. The A allele of rs1990760 and the A-G (rs1990760/rs3747517) haplotype are highly correlated with the risk of PsO. However, the A allele and A-G haplotype of rs1990760 have been identified as protective factors for chronic periodontitis. This study also confirmed the hypothesis that there are shared molecular mechanisms from common genetic variations which collectively lead to a series of immune-related diseases.

### 4.2 Common or Differential Genetic Pathogenesis

A large number of current literature indicate that there is a concomitant relationship between PsO and cardiometabolic diseases, gastrointestinal diseases, malignant tumors, infections, and mood disorders. However, the intrinsic connection between PsO and the coexistence of comorbidities is not clear. Shared inflammatory pathways, cellular mediators, genetic susceptibility, and common risk factors are currently the most popular proposed mechanisms (Takeshita et al., 2017). Through genetic studies, researchers have not only explored the existence of some common or differential susceptibility genes between PsO and its comorbidities but have also explored whether there is a common genetic pathogenesis.

Sundarrajan and Arumugam (2016) used an unbiased integrated network medical method to study the biological processes and pathways of PsO and five common comorbidities (myocardial infarction, T2DM, obesity, RA, and Alzheimer’s disease). It was observed that there is a clear overlap between genes acting in the same direction in PsO and its comorbidities, which proves the inevitability of one of the comorbidities (Sundarrajan and Arumugam, 2016). The biological processes involving inflammation and cell signal transduction form the common basis between PsO and its related comorbidities. Pathway analysis revealed several common pathways (e.g., angiogenesis) and rare pathways (e.g., CCKR signal diagram and gonadotropin realization hormone receptor pathway) that overlap in PsO and all five comorbidities. This study revealed some previously overlooked common genes and pathways and provided evidence for the hypothesis that there are shared components between PsO and its comorbidities.

GWAS identified that TRAF3IP2 is the common susceptibility gene of PsO and PsA (Hüffmeier et al., 2010). Functional studies showed that the TRAF3IP2 variant (p.Asp10Asn, rs33980500) reduced the binding of TRAF6, indicating that by changing the interaction of TRAF as a new shared pathway between PsA and PsO, the regulation of immune regulatory signals was altered. Aterido et al. (2019) performed the GWAS on PsA patients and utilized controls from a Spanish population and detected genetic associations at the individual marker and pathway levels. Furthermore, this group performed a meta-analysis on patients from North America and highlighted 14 genetic pathways that are significantly related to PsA. By comparing the PsA cohort with the PsC and RA cohorts, it was confirmed that the glycosaminoglycan (GAG) metabolic pathway is a specific pathway for PsA.

Chen et al. (2019) explored the common pathogenesis between PsA, PsV, RA, and gouty arthritis (GA) and determined that these diseases co-express plasma exosomal microRNA. By evaluating the expression ranking of PsA, PsV, RA, and GA and the plasma exosomes of healthy controls, a total of 36 commonly expressed microRNAs were detected in patients with PsA, PsV, RA, and GA. Gene Ontology (GO) and Kyoto Encyclopedia of Genes and Genomes (KEGG) were used to carry out microRNA enrichment analysis of common expressions. Among the 36 commonly expressed microRNAs, there are five microRNAs (hsa-miR-151a-3p, hsa-miR-199a-5p, hsa-miR-370-3p, hsa-miR-589-5p, and hsa-miR-769-5p) that are considered to be related to the common pathogenesis of PsA, PsV, RA, and GA. A systematic review shows that the effects of these five microRNAs are related to immune dysfunction and bone damage, which is consistent with the conclusions of GO and KEGG analyses.

Zheng et al. (2016) explored the potential association between RA and three autoimmune diseases [PsO, type 1 diabetes mellitus (T1DM), and SLE]. First, a comprehensive excavation of public databases was conducted to discover risk genes related to RA, T1DM, SLE, and PsO. Then, the risk pathways related to the disease were explored through the enrichment analysis of these genes to identify common pathways shared by RA and the other three diseases. A variety of RA risk pathways with obvious pleiotropic effects have been identified, specifically being immunology-related pathways. At the same time, it was emphasized that the viral myocarditis pathway associated with CVD is involved in autoimmune diseases. These results indicate that RA and these three autoimmune diseases (PsO, T1DM, and SLE) share common genetic risk components, causing individuals to be susceptible to autoimmune diseases.

GO and network analyses showed (Baurecht et al., 2015) that the GO Term “keratinocyte differentiation” is rich in genes related to the risk of AD and PsO. Additionally, the GO term “response to interferon-γ” demonstrated enriched genes related to risk of PsO. It shows that AD and PsO have unique genetic mechanisms that affect the shared pathways of epidermal differentiation and immune response. Ito et al. (2004) used DNA chips to comprehensively analyze mRNA expression in skin biopsy tissues of AD or PsO patients, and then used quantitative PCR to monitor the expression of new disease-related genes in human keratinocytes or auricles from NC/Nga mice. Compared with the unaffected skin, there was an increase in IDO (indoleamine 2,3-dioxygenase) and kynurenase (enzyme that constitutes the tryptophan degradation pathway) mRNA levels in the skin lesions of AD or PsO. In addition, the expression of canine urokinase mRNA in ear skin was induced after AD-like skin lesions occurred in NC/Nga mice. This study indicates that the tryptophan degradation pathway may play an important role in the pathophysiology of AD and PsO.

In order to determine the candidate causal SNPs and candidate causal mechanism of PsO and Behcet’s disease (BD), a hypothesis was formed in the following fashion: SNP→gene→pathway. Lee et al. (2012) attempted to identify candidate causal SNPs and pathways (ICSN pathway) and analyzed PsO and BD GWAS datasets. This led to the identification of candidate pathogenic SNPs and candidate pathways that may lead to PsO and BD susceptibility. ICSN pathway analysis identified fifteen candidate causal SNPs and 28 candidate causal pathways (Lee et al., 2012). The top five significant candidate causal SNPs are rs1063478, rs8084, rs7192, rs20541, and rs1130838, all of which are located at the HLA locus except for SNP rs20541 (IL-13). These candidate causal SNPs and pathways provide ten hypothetical biological mechanisms, and the most closely related pathway involves the HLA locus. When the HLA locus is excluded, the ICSN pathway analysis provides the following hypothetical biological mechanisms: rs20541 (non-synonymous code) → IL-13 → dendritic cells are involved in the development of Th1 and Th2 and the regulation of the GATA3 pathway.

In addition, researchers have also carried out studies on the possible shared pathways between PsO and some other comorbidities as follows: a) to explore the common pathogenesis between PsO and DM, Wang et al. (2017) genotyped 89 DM susceptibility loci in Chinese PsO patients and healthy controls. This allowed for a pathway analysis, with results suggesting that the T-cell receptor signaling pathway is involved in the pathogenesis of PsO. Next, b) the role of IL-36 family cytokines has been determined in the pathogenesis of PsO. Although there may be a significant mechanical overlap between PsO and IBD, studies have shown that there is an increase in the expression level of IL-36α in the colonic mucosa of UC patients. The new role of IL-36 signaling in colon inflammation has been determined through experiments in animal models, indicating that the IL-36 pathway may provide a new target for the treatment and intervention of IBD (Russell et al., 2016). Last, c) in order to explore the potential shared biological process between PsO and SCZ, Yin et al. (2016) conducted GO analysis using the datasets of SCZ, PsO, and their combined populations (PsO and SCZ combined). The authors found that the shared genes are mainly involved in antigen processing, and it is enriched in pathways related to presentation and endoplasmic reticulum.

## 5 Conclusion and Perspectives

Current epidemiological studies have reported many comorbidities of PsO, and some common susceptibility genes and shared genetic pathogenesis between PsO and its comorbidities have been found. Further understanding of PsO and its comorbidities will promote research on the pathogenesis, drug development, novel therapy methods, and personalized and precision treatment of PsO and its comorbidities. However, to truly clarify the intrinsic correlation between PsO and its comorbidities, further experimental research is necessary. In addition, physicians should be cognizant and screen for the comorbidities of PsO in the clinic. Comorbidities should be taken into account when considering the prevention, treatment, and prognosis of PsO. This will lead into a more comprehensive diagnosis and treatment of PsO as a whole, which is beneficial to recovery and management of PsO.
