# Supplementary material for: Cognitive Process of Psoriasis and Its Comorbidities: From Epidemiology to Genetics
Source: Front Genet. 2021 Nov 26;12:735124. doi: 10.3389/fgene.2021.735124 (PMC8662384; doi:10.3389/fgene.2021.735124)
Supplement: Supplementary file 1 [file DataSheet1.docx]

Supplementary Table S1 Reported comorbidities of psoriasis in forms of case report and systematic review

| **Comorbidities** | **Description** | **Rresearch type** | **Ref.** |
| --- | --- | --- | --- |
| Syphilis | 1 case | Case report | ([1](#_ENREF_1)) |
| Linear lichen planus | 1 case | Case report | ([2](#_ENREF_2)) |
| Leprosy | 1 case | Case report | ([3](#_ENREF_3)) |
| Dermatomyositis | 1 case | Case report | ([4](#_ENREF_4)) |
| Alopecia areata | 1 case | Case report | ([5](#_ENREF_5)) |
| T cell leukemia | 1 case | Case report | ([6](#_ENREF_6)) |
| Prolactinoma | 3 cases | Case report | ([7](#_ENREF_7)) |
| Neurofibromatosis | 1 case | Case report | ([8](#_ENREF_8)) |
| Myeloid leukemia | 1 case | Case report | ([9](#_ENREF_9)) |
| Chronic myeloid leukemia | 1 case | Case report | ([10](#_ENREF_10)) |
| Bowen disease | 1 case | Case report | ([11](#_ENREF_11)) |
| Bladder cancer | 1 case | Case report | ([12](#_ENREF_12)) |
| B cell lymphoma | 1 case | Case report | ([13](#_ENREF_13)) |
| Epilepsy | 1 case | Case report | ([14](#_ENREF_14)) |
| Multiple osteomyelitis | 2 cases | Case report | ([15](#_ENREF_15)) |
| Cryptococcal meningitis | 1 case | Case report | ([16](#_ENREF_16)) |
| Acquired immune deficiency syndrome | 2 cases | Case report | ([17](#_ENREF_17)) |
| Primary biliary cirrhosis | 6 cases | Case report | ([18](#_ENREF_18)) |
| Liver cirrhosis | 4 cases | Case report | ([19](#_ENREF_19)) |
| Sjögren's syndrome | 5 cases | Case report | ([20](#_ENREF_20)) |
| Rheumatoid arthritis | 1 case | Case report | ([21](#_ENREF_21)) |
| Pigment dissemination syndrome | 2 cases | Case report | ([22](#_ENREF_22)) |
| Cataract | 5 cases | Case report | ([23](#_ENREF_23)) |
| Lupus nephritis | 1 case | Case report | ([24](#_ENREF_24)) |
| Cushing syndrome | 1 case | Case report | ([25](#_ENREF_25)) |
| Antisynthetase syndrome | 1 case | Case report | ([26](#_ENREF_26)) |
| Behcet's disease | 9 cases | Case report | ([27](#_ENREF_27)) |
| Pruritus | 80-100% PsO patients have pruritus | Systematic review | ([28](#_ENREF_28)) |
| Chronic spontaneous urticaria | Incidence of PsO in chronic spontaneous urticaria ≥ 1% | Systematic review | ([29](#_ENREF_29)) |
| Uveitis | 7-20% PsO patients have uveitis | Systematic review | ([30](#_ENREF_30)) |
| Obstructive sleep apnea | 36-81.8% PsO patients have obstructive sleep apnea | Systematic review | ([31](#_ENREF_31)) |

Supplementary Table S2 Summarization of epidemiological studies between psoriasis and its comorbidities (tumors or cancers)*

| **Comorbidities** | **Description** | **Rresearch type** | **Ref.** |
| --- | --- | --- | --- |
| Sarcoidosis | Of the 443 patients identified with both PsO and sarcoidosis by database search, 68 individuals had both diagnoses confirmed by manual chart review | Observational cross-sectional study | ([32](#_ENREF_32)) |
| Respiratory tract cancer | 37 articles were selected in this meta-analysis. PsO patients have increasing risk of respiratory tract cancer, upper aerodigestive tract cancer, urinary tract cancer, liver cancer, squamous cell carcinoma, basal cell carcinoma, and Non-Hodgkin's lymphoma, the SIR and 95% CI were :1.52 (1.35-1.71), 3.05 (1.74-5.32), 1.31 (1.11-1.55), 1.90 (1.48-2.44), 5.3 (2.63-10.71), 2.00 (1.83-2.20), 1.40 (1.06-1.86), respectively | Meta-analysis | ([33](#_ENREF_33)) |
| Upper aerodigestive tract cancer |  | Meta-analysis | ([33](#_ENREF_33)) |
| Urinary tract cancer |  | Meta-analysis | ([33](#_ENREF_33)) |
| Liver cancer |  | Meta-analysis | ([33](#_ENREF_33)) |
| Squamous cell carcinoma |  | Meta-analysis | ([33](#_ENREF_33)) |
| Basal cell carcinoma |  | Meta-analysis | ([33](#_ENREF_33)) |
| Non-Hodgkin's Lymphoma |  | Meta-analysis | ([33](#_ENREF_33)) |
| Lung cancer | A total of 937,716 control group patients without PsO, and 198,366 patients with PsO were included in the analysis. PsO patients have higher incidences of lung cancer (aHR = 1.15, 95% CI: 1.03-1.27) and lymphoma (aHR = 1.34, 95% CI: 1.18-1.51) compare with controls, respectively | Cohort study | ([34](#_ENREF_34)) |
| Lymphoma |  | Cohort study | ([34](#_ENREF_34)) |
| Hodgkin's lymphoma | 153,197 patients with PsO and 765,950 corresponding subjects without PsO were included. PsO patients who received a systemic treatment consistent with extensive disease were classified as severe (n = 3,994) and those who did not receive systemic therapies were classified as mild (n = 149,203). Mild and severe PsO patients have higher incidence of Hodgkin's lmphoma (mild: aHR = 1.42, 95% CI: 1.00-2.02; severe: aHR = 3.18, 95% CI: 1.01-9.97) compare with controls | Cohort study | ([35](#_ENREF_35)) |
| Breast cancer | 599 individuals were diagnosed as PsO in 56,235 breast cancer patients; breast cancer patients have increasing risk of PsO (HR = 1.17, 95% CI: 1.07-1.28) | Cross-sectional study | ([36](#_ENREF_36)) |
| Colorectal cancer | 9 cohort studies with 10,544,609 subjects were included in this meta-analysis. A significantly increased risk of colorectal cancer in patients with PsO (HR = 1.16, 95% CI: 1.08-1.24) | Meta-analysis | ([37](#_ENREF_37)) |
| Pancreatic cancer | Among 67,761 patients from the UK General Practice Research Database, 1,703 patients had incident cancer; of whom 54% had a history of PsO. PsO patients have higher incidences of pancreatic cancer (IRR = 2.20, 95% CI: 1.18-4.09) compare with controls | Case-control study | ([38](#_ENREF_38)) |

*There is more than one study about PsO and its comorbidities that have been conducted. Here, we have listed the study with a bigger sample size and refer to the evidence level.

Note: RR: risk ratio; HR: hazard ratio; aHR: adjusted hazard ratio; SIR: standardized incidence ratio; IRR: incidence rate ratio; CI: confidence interval.

Supplementary Table S3 Summarization of epidemiological studies between psoriasis and its comorbidities (cardiovascular diseases)*

| **Comorbidities** | **Description** | **Rresearch type** | **Ref.** |
| --- | --- | --- | --- |
| Aortic stenosis | A total of 5,107,624 subjects were eligible for analysis, there were 58,747 patients with mild PsO and 11,918 patients with severe PsO. PsO patients have increasing risk of aortic stenosis (mild: IRR = 1.22, 95% CI: 1.12-1.33; severe: IRR = 1.61, 95% CI: 1.32-1.96) | Cohort study | ([39](#_ENREF_39)) |
| Cardiovascular diseases | Of the 14 included studies, 10 were population-based cohorts, and sample sizes in the PsO group ranged from 462 to 130,976. Severe PsO with higher incidence of cardiovascular diseases (RR = 1.37, 95% CI: 1.17-1.60), myocardial infarction (RR = 3.04, 95% CI: 0.65-14.35), stroke (RR = 1.59, 95% CI: 1.34-1.89) compare with controls, respectively | Meta-analysis | ([40](#_ENREF_40)) |
| Myocardial infarction |  | Meta-analysis | ([40](#_ENREF_40)) |
| Stroke |  | Meta-analysis | ([40](#_ENREF_40)) |
| Pulmonary hypertension | There were 10,115 patients with mild PsO, 3821 with severe PsO, and 69,360 matched controls in this study. Severe PsO patients have increasing risk of pulmonary hypertension (HR = 1.46, 95% CI: 1.09-1.94) compare with controls | Retrospective cohort study | ([41](#_ENREF_41)) |
| Peripheral vascular disease | A total of 3,236 patients with PsO and 2,500 controls were identified in the Miami VA Medical Center electronic database. PsO patients have higher incidence of peripheral vascular disease (OR = 1.98, 95% CI: 1.32-2.82) and ischemic heart disease (OR = 1.78, 95% CI: 1.51-2.11) compare with controls, respectively | Observational study | ([42](#_ENREF_42)) |
| Ischemic heart disease |  | Observational study | ([42](#_ENREF_42)) |
| Coronary artery calcification | 159 control subjects and 190 PsO patients included in this study, PsO patients included in the evaluation of coronary artery calcification were 152. The incidence of coronary artery calcification in PsO patients was 35.5% (54/152) | Observational study | ([43](#_ENREF_43)) |
| Atrial fibrillation | 3 retrospective studies with 110,568 cases of PsO and 5,352,817 participants without PsO were included in this meta-analysis. The incidence of atrial fibrillation in PsO patients is higher than in no-PsO individuals (RR = 1.21, 95% CI: 1.14-1.29) | Meta-analysis | ([44](#_ENREF_44)) |
| Hypertension | 24 observational studies with a total of approximately 2.7 million participants were included. Of them, 309,469 were patients with PsO. PsO patients with increasing ratio of hypertension compare with controls (OR = 1.58, 95% CI: 1.42-1.76) | Cross-sectional study | ([45](#_ENREF_45)) |
| Heart failure | A total of 5,485,856 subjects were eligible for analysis. In the study period, 66,389 patients with new-onset PsO, including 11,242 patients with severe PsO, were identified. The incidence of heart failure in the control group, mild PsO, and severe PsO were 2.82, 4.22, and 4.70 per thousand people per year | Cohort study | ([46](#_ENREF_46)) |
| Coronary heart disease | 113,065 in-hospital and clinic patients were included. The incidence of coronary heart disease in PsO patients was 9.0%, multivariate analysis showed that PsO was an independent risk factor for coronary heart disease (OR = 1.27, 95% CI: 1.01-1.58) | Retrospective study | ([47](#_ENREF_47)) |
| Coronary artery disease | 14 eligible studies provided data on 1,427 patients with PsO and 9,670 controls. PsO patients with increasing ratio of coronary artery disease (RR = 1.14, 95% CI: 1.04-1.26) | Meta-analysis | ([48](#_ENREF_48)) |

*There is more than one study about PsO and its comorbidities that have been conducted. Here, we listed the study with a bigger sample size and refer to the evidence level.

Note: OR: odds ratio; RR: risk ratio; IRR: incidence rate ratio; HR: hazard ratio; CI: confidence interval.

Supplementary Table S4 Summarization of epidemiological studies between psoriasis and its comorbidities (neuropsychiatric disorders)*

| **Comorbidities** | **Description** | **Rresearch type** | **Ref.** |
| --- | --- | --- | --- |
| Schizophrenia | A total of 5 studies with more than 6 million participants met the eligibility criteria and were included in this meta-analysis. PsO patients with increasing ratio of schizophrenia than no-PsO groups (total OR = 1.41, 95% CI: 1.19-1.66) | Meta-analysis | ([49](#_ENREF_49)) |
| Insanity | 31 studies, comprising data for >25 million individuals, were eligible. PsO associate with insanity (OR = 1.70, 95% CI: 1.51-1.91) | Observational study | ([50](#_ENREF_50)) |
| Parkinson's disease | 4 study met eligibility criteria and were included in this meta-analysis. PsO patients with increasing risk of Parkinson's disease than controls (RR = 1.38, 95% CI: 1.15-1.66) | Meta-analysis | ([51](#_ENREF_51)) |
| Depressive symptoms | In the meta-analysis of the 98 studies, data were pooled on 401,703 PsO patients. More than 10% PsO patients have clinical depressive symptoms | Meta-analysis | ([52](#_ENREF_52)) |
| Depression | This study included 17,086 patients with PsO and 1,607,242 patients from the general population. Incidence of depression in PsO patients was 11.52%, while the incidence was 7.73% in controls | Cross-sectional study | ([53](#_ENREF_53)) |
| Insomnia | Participants included 179 patients with plaque PsO and 105 controls. 25% PsO patients were reported with insomnia, the incidence was 10.5% in controls | Case-control study | ([54](#_ENREF_54)) |
| Anxiety | 938,194 patients from 15 papers were included. 7-48% PsO patients have anxiety, significantly higher than health controls (OR = 2.91, 95% CI: 2.01-4.21) | Observational study | ([55](#_ENREF_55)) |

*There is more than one study about PsO and its comorbidities that have been conducted. Here, we listed the study with a bigger sample size and refer to the evidence level.

Note: OR: odds ratio; RR: risk ratio; CI: confidence interval.

Supplementary Table S5 Summarization of epidemiological studies between psoriasis and its comorbidities (diseases of the digestive system)*

| **Comorbidities** | **Description** | **Rresearch type** | **Ref.** |
| --- | --- | --- | --- |
| Ulcerative colitis | 13 studies reported data on the prevalence of ulcerative colitis in 396,049 patients with PsO, the incidence of ulcerative colitis in PsO patients is 0.5% (95% CI: 0.3-0.8%) | Meta-analysis | ([56](#_ENREF_56)) |
| Crohn's disease | 17 studies reported on the occurrence of crohn's disease in 481,536 PsO patients, the incidence of Crohn's disease in PsO patients is 0.7% (95% CI: 0.2-1.3%) | Meta-analysis | ([56](#_ENREF_56)) |
| Liver fibrosis | 400 adults with severe PsO were recruited, 333 had a successful transient elastography scan and were included in final analysis. 47 patients with moderate PsO were diagnosed with liver fibrosis | Prospective cohort study | ([57](#_ENREF_57)) |
| Helicobacter pylori infection | 9 observational studies involving 1546 individuals were included. PsO patients have a higher incidences of helicobacter pylori infection than controls (OR = 1.58, 95% CI: 1.02-2.46） | Meta-analysis | ([58](#_ENREF_58)) |
| Celiac disease | 4 retrospective cohort studies with 12,912 cases of PsO and 24,739 comparators were included in this meta-analysis. PsO patients have a higher incidence of celiac disease than controls (OR = 3.09, 95% CI: 1.92-4.97) | Meta-analysis | ([59](#_ENREF_59)) |
|  | 18 studies were included. Random effects meta-analysis found significant OR of 2.16 (95% CI: 1.74-2.69; 9 studies) for celiac disease in patients with PsO and 1.8 (95% CI: 1.36-2.38) for PsO in patients with celiac disease | Meta-analysis | ([60](#_ENREF_60)) |

*There is more than one study about PsO and its comorbidities that have been conducted. Here, we listed the study with a bigger sample size and refer to the evidence level.

Note: OR: odds ratio; CI: confidence interval.

Supplementary Table S6 Summarization of epidemiological studies between psoriasis and its comorbidities (metabolic diseases)*

| **Comorbidities** | **Description** | **Rresearch type** | **Ref.** |
| --- | --- | --- | --- |
| Metabolic syndrome | 63 studies encompassing 15,939 PsO patients and 103,984 controls were included in this meta-analysis. 30.29% PsO patients were reported with metabolic syndrome (21.70% in controls); increasing incidence of metabolic syndrome in PsO patients (OR = 2.077, 95% CI: 1.84-2.34) | Meta-analysis | ([61](#_ENREF_61)) |
| Hashimoto's thyroiditis | 41 patients with PsO and Hashimoto's thyroiditis: PsO significantly associate with Hashimoto's thyroiditis (OR = 2.49, 95% CI: 1.79-3.48) | Retrospective cross-sectional study | ([62](#_ENREF_62)) |
|  | The prevalence of Hashimoto's thyroiditis in PsO was 21.6%; in the controls, it was 6.6%. In patients with PsO, Hashimoto's thyroiditis was more common in women and less common in those who had polyarticular arthropathic PsO and plaque PsO. | Cross-sectional observational study | ([63](#_ENREF_63)) |
| Gout | A total of 114,623 patients with gout and 114,623 patients without gout were identified in this study. Gout was significantly associated with PsO (aOR = 1.30, 95% CI: 1.20-1.42) | Cross-sectional study | ([64](#_ENREF_64)) |
| Diabetes mellitus | 38 studies including 922,870 cases with PsO and 12,808,071 controls entered the meta- analysis. PsO was significantly associated with diabetes mellitus (OR = 1.69, 95% CI: 1.51-1.89) | Meta-analysis | ([65](#_ENREF_65)) |
| Abdominal obesity | The total population consisted of 22,633,536 subjects, among whom 399,461 had newly developed PsO. Abdominal obesity males have higher risk of PsO than controls (HR = 1.175, 95% CI: 1.15-1.20) | Prospective cohort study | ([66](#_ENREF_66)) |

*There is more than one study about PsO and its comorbidities that have been conducted. Here, we listed the study with a bigger sample size and refer to the evidence level.

Note: OR: odds ratio; aOR: adjusted odds ratio; HR: hazard ratio; CI: confidence interval.

Supplementary Table S7 Summarization of epidemiological studies between psoriasis and its comorbidities (other diseases)*

| **Comorbidities** | **Description** | **Rresearch type** | **Ref.** |
| --- | --- | --- | --- |
| Temporomandibular joint disorder | 112 patients with PsO and 112 subjects without PsO were recruited in this study. 44.3% of PsO patients showed temporomandibular joint disorder versus 22.3% of controls | Observational study | ([67](#_ENREF_67)) |
| Restless legs syndrome | 300 patients with PsO and 300 healthy controls were recruited in this study. 17% PsO patients have restless legs syndrome, only 4% no-PsO individuals have restless legs syndrome | Case-control study | ([68](#_ENREF_68)) |
| Erectile dysfunction | 9 studies with 36,242 PsO patients and 1,657,711 controls met inclusion criteria in this meta-analysis. PsO significantly associated with the risk of erectile dysfunction (OR = 1.35, 95% CI: 1.29-1.41) | Meta-analysis | ([69](#_ENREF_69)) |
| Periodontitis | 5 studies (2 cohort studies and 3 case-control studies) with 312,584 subjects met the eligibility criteria were included in the meta-analysis. Increasing risk of PsO in periodontitis patients compare with controls (RR = 1.55, 95% CI: 1.35-1.77) | Meta-analysis | ([70](#_ENREF_70)) |
| Fracture | 9,788 patients with psoriatic arthritis, 158,323 patients with PsO, and 821,834 matched controls were identified. Mild PsO patients have a higher risk of fracture (of all types), vertebral and hip fractures with aHR of 1.07 (95% CI: 1.05-1.10), 1.17 (95% CI: 1.03-1.33), and 1.13 (95% CI: 1.04-1.22), respectively；severe PsO patients have a significantly increased risk of all fractures and vertebral fractures, with aHR of 1.26 (95% CI: 1.15-1.39) and 2.23 (95% CI: 1.54-3.22), respectively | Cohort study | ([71](#_ENREF_71)) |
| Osteoporosis | 17,507 cases with osteoporosis and 52,521 controls without a history of osteoporosis were included in this study. There is a higher incidence of PsO in osteoporosis patients than controls (1.50% vs. 0.87%) | Case-control study | ([72](#_ENREF_72)) |
| Multiple sclerosis | A total of 11 studies were included, included 43,643 multiple sclerosis patients and 1,097,374  controls. PsO associated with multiple sclerosis (OR = 1.29, 95% CI: 1.14-1.45) and increase the risk of PsO (HR = 1.92, 95% CI: 1.32-2.80) | Meta-analysis | ([73](#_ENREF_73)) |
| IgA nephropathy | 205,815 PsO patients (mild: 193,013; moderate-to-severe: 12,806) and 1,019,140 patients without PsO were identified. moderate to severe PsO patients were significantly associated with the risk of IgA nephropathy (HR = 4.75, 95% CI: 1.92–11.76) and glomerular disease (HR = 2.05, 95% CI: 1.10–3.84), respectively | Cohort study | ([74](#_ENREF_74)) |
| Glomerular disease |  | Cohort study | ([74](#_ENREF_74)) |
| Chronic obstructive pulmonary disease | 4 observational studies with a total of 13,418 subjects were included in this study. The incidence of chronic obstructive pulmonary disease in PsO patients is higher than controls (total OR = 1.45, 95% CI: 1.21-1.73) | Meta-analysis | ([75](#_ENREF_75)) |
| Chronic kidney disease | A total of 4 retrospective cohort studies with 199,808 patients with PsO were included. Increasing risk of chronic kidney disease (total RR = 1.34, 95% CI: 1.14-1.57) and end-stage renal disease in PsO patients (total RR = 1.29, 95% CI: 1.05-1.60) in PsO patients compare with controls, respectively | Meta-analysis | ([76](#_ENREF_76)) |
| End-stage renal disease |  | Meta-analysis | ([76](#_ENREF_76)) |
| Hyperhidrosis | The database included 887,765 adolescents, of whom 3,112 (0.35%) were diagnosed with PsO. Hyperhidrosis was significantly associated with PsO (OR = 1.51, 95% CI: 1.21-1.86) | Cross-sectional study | ([77](#_ENREF_77)) |
| Asthma | A total of 6 studies with 66,772 PsO cases and 577,415 controls were included. PSO was significantly associated with an increased risk of asthma (OR = 1.32, 95% CI: 1.20-1.46) | Meta-analysis | ([78](#_ENREF_78)) |
| Systemic sclerosis | There were 150 (1.2%) cases of PsO among controls and 47 (1.9%) among systemic sclerosis -patients. A systemic sclerosis diagnosis was an independent risk factor for PsO with an OR of 2.16 (95% CI: 1.38-3.39) | Cohort study | ([79](#_ENREF_79)) |
| Oral mucosal damage | Oral mucosal lesions were diagnosed in 43 (43%) psoriatic patients and 17 (17%) control subjects | Case-control study | ([80](#_ENREF_80)) |

*There is more than one study about PsO and its comorbidities that have been conducted. Here, we listed the study with a bigger sample size and refer to the evidence level.

Note: OR: odds ratio; RR: risk ratio; HR: hazard ratio; aHR: adjusted hazard ratio; CI: confidence interval.

**REFERENCES**

[1] Kase K, Ishii-Osai Y, Sumikawa Y, Yoneta A, Himeno D, Kakutani Y, et al. Rapidly developed neurosyphilis in a psoriasis patient under treatment with infliximab: a case report. Acta Derm Venereol. (2015) 95(3): 351-2. doi: 10.2340/00015555-1932

[2] Ohshima N, Shirai A, and Asahina A. Coexistence of linear lichen planus and psoriasis in a single patient. J Dermatol. (2011) 38(12): 1182-4. doi: 10.1111/j.1346-8138.2010.01188.x

[3] Raiol TK, Volpato SE, Santana JM, Ferreira IS, and Takano DM. Leprosy associated with psoriasis. Lepr Rev. (2015) 86(4): 368-73. doi: 10.47276/lr.86.4.368

[4] Xing Y, Xie J, Jiang S, Upasana M, and Song J. Co-existence of Juvenile dermatomyositis and psoriasis vulgaris with fungal infection: A case report and literature review. J Cosmet Dermatol. (2019) 18(5): 1560-3. doi: 10.1111/jocd.12869

[5] Khaled A, Hawilo A, Zaouak A, Zeglaoui F, Kharfi M, and Kamoun MR. Association between scalp psoriasis and alopecia areata. Tunis Med. (2012) 90(4): 344. PMID: 22535354.

[6] Koga M, Koga K, Isitsuka K, Imafuku S, and Nakayama J. Coexistence of adult T-cell leukaemia /lymphoma and psoriasis treated with cyclosporine. Eur J Dermatol. (2012) 22(2): 275-6. doi: 10.1684/ejd.2011.1637

[7] Sanchez Regana M, and Umbert Millet P. Psoriasis in association with prolactinoma: three cases. Br J Dermatol. (2000) 143(4): 864-7. doi: 10.1046/j.1365-2133.2000.03792.x

[8] Vasili E, Saraceno R, Vargu M, Hysi K, Kellici S, and Fida M. Neurofibromatosis associated with plaque-type psoriasis: coincidental occurrence or causal association? Cutis. (2012) 90(3): 147-8. doi: 10.1001/archdermatol.2012.2080

[9] Gu Y, Li K, Zhou Y, and Zhang J. Chronic myeloid leukaemia in a patient with psoriasis following bimolane treatment. J Eur Acad Dermatol Venereol. (2020) 34(2): e66-67. doi: 10.1111/jdv.15915

[10] Nagai T, Karakawa M, Komine M, Muroi K, Ohtsuki M, and Ozawa K. Development of psoriasis in a patient with chronic myelogenous leukaemia during nilotinib treatment. Eur J Haematol. (2013) 91(3): 270-2. doi: 10.1111/ejh.12153

[11] David M, Grunwald M, and Feuerman EJ. Bowen's disease in a patient with psoriasis vulgaris. Harefuah. (1982) 102(7): 282-3. doi: 10.1097/00004872-201106001-01621

[12] Ruzzetti M, Saraceno R, Fabiano S, and Chimenti S. Psoriasis disappearance after resection of a bladder tumour. Eur J Dermatol. (2010) 20(5):646-7. doi: 10.1684/ejd.2010.1033

[13] Bardazzi F, Antonucci VA, Alessandrini AM, Baraldi C, Tengattini V, and Patrizi A. B-cell lymphoma in a psoriatic patient treated with infliximab. Eur J Dermatol. (2013) 23(2): 264-5. doi: 10.1684/ejd.2013.1925

[14] Bartsch T, Rempe T, Wrede A, Leypoldt F, Bruck W, Adams O, et al. Progressive neurologic dysfunction in a psoriasis patient treated with dimethyl fumarate. Ann Neurol. (2015) 78(4): 501-14. doi: 10.1002/ana.24471

[15] Laxer RM, Shore AD, Manson D, King S, Silverman ED, and Wilmot DM. Chronic recurrent multifocal osteomyelitis and psoriasis--a report of a new association and review of related disorders. Semin Arthritis Rheum. (1988) 17(4): 260-70. doi: 10.1016/0049-0172(88)90011-x

[16] Cainelli F, Concia E, and Vento S. Cryptococcal meningitis during cyclosporin treatment in a patient with psoriasis. Br J Dermatol. (2000) 143(6): 1327-8. doi: 10.1046/j.1365-2133.2000.03916.x

[17] Blanco Gonzalez OA, Larrondo Muguercia RJ, Blanco Gonzalez BL, and Rodriguez Barreras ME. Psoriasis and AIDS: a report of 2 cases. Rev Cubana Med Trop. (2000) 52(5): 148-9. PMID: 11107911.

[18] Ohira H, Rai T, Takiguchi J, Abe K, and Sato Y. Six cases of primary biliary cirrhosis complicated by psoriasis. Hepatol Res. (2004) 30(2): 111-5. doi: 10.1016/j.hepres.2004.06.003

[19] Ergun T, Seckin-Gencosmanoglu D, Salman A, Ozgen Z, Ocak ES, Avsar E, et al. Tumor necrosis factor-alpha inhibitors for the treatment of psoriasis patients with liver cirrhosis: A report of four cases with a literature review. Indian J Dermatol Venereol Leprol. (2017) 83(1): 55-9. doi: 10.4103/0378-6323.186498

[20] Yamamoto T, Katayama I, and Nishioka K. Overlapping cases with psoriasis and Sjogren syndrome: a study of lymphocyte response to staphylococcal enterotoxin B. J Dermatol Sci. (1996) 13(3): 212-8. doi: 10.1016/s0923-1811(96)00537-3

[21] Toussirot E. New onset of psoriasis in a patient with rheumatoid arthritis treated with rituximab. J Rheumatol. (2013) 40(7): 1230-1. doi: 10.3899/jrheum.130050

[22] Evereklioglu C, Inaloz HS, and Kirtak N. Psoriasis with pigment dispersion syndrome: report of two cases. J Eur Acad Dermatol Venereol. (2003) 17(4): 488-9. doi: 10.1046/j.1468-3083.2003.00614_14.x

[23] Wanscher B, and Vesterdal E. Syndermatotic cataract in patients with psoriasis. Acta Derm Venereol. (1976) 56(5): 397-9. PMID: 78627.

[24] Satoh Y, Nakano K, Yoshinari H, Nakayamada S, Iwata S, Kubo S, et al. A case of refractory lupus nephritis complicated by psoriasis vulgaris that was controlled with secukinumab. Lupus. (2018) 27(7): 1202-6. doi: 10.1177/0961203318762598

[25] Eichhoff G. Tinea Incognito Mimicking Pustular Psoriasis in a Patient With Psoriasis and Cushing Syndrome. Cutis. (2021) 107(4): E30-32. doi: 10.12788/cutis.0239

[26] Hanami Y, Kumekawa M, and Yamamoto T. Psoriasis vulgaris in a patient with antisynthetase syndrome. J Dermatol. (2021) 48(1): e39-40. doi: 10.1111/1346-8138.15636

[27] Cho S, Cho SB, Choi MJ, Zheng Z, and Bang D. Behçet's disease in concurrence with psoriasis. J Eur Acad Dermatol Venereol. (2013) 27(1): e113-8. doi: 10.1111/j.1468-3083.2012.04559.x

[28] Therene C, Brenaut E, Barnetche T, and Misery L. Efficacy of Systemic Treatments of Psoriasis on Pruritus: A Systemic Literature Review and Meta-Analysis. J Invest Dermatol. (2018) 138(1): 38-45. doi: 10.1016/j.jid.2017.05.039

[29] Kolkhir P, Borzova E, Grattan C, Asero R, Pogorelov D, and Maurer M. Autoimmune comorbidity in chronic spontaneous urticaria: A systematic review. Autoimmun Rev. (2017) 16(12): 1196-1208. doi: 10.1016/j.autrev.2017.10.003

[30] Fotiadou C, and Lazaridou E. Psoriasis and uveitis: links and risks. Psoriasis (Auckl). (2019) 9: 91-6. doi: 10.2147/ptt.S179182

[31] Gupta MA, Simpson FC, and Gupta AK. Psoriasis and sleep disorders: A systematic review. Sleep Med Rev. (2016) 29: 63-75. doi: 10.1016/j.smrv.2015.09.003

[32] Wallace EB, Khosravi H, Joyce CJ, Vleugels FR, and Patel M. Examining the epidemiology of coincident psoriasis and sarcoidosis: An observational cross-sectional study. J Am Acad Dermatol. (2018) S0190-9622(18)32893-7. doi: 10.1016/j.jaad.2018.11.009

[33] Pouplard C, Brenaut E, Horreau C, Barnetche T, Misery L, Richard MA, et al. Risk of cancer in psoriasis: a systematic review and meta-analysis of epidemiological studies. J Eur Acad Dermatol Venereol. (2013) 27 Suppl 3: 36-46. doi: 10.1111/jdv.12165

[34] Chiesa Fuxench ZC, Shin DB, Ogdie Beatty A, and Gelfand JM. The Risk of Cancer in Patients With Psoriasis: A Population-Based Cohort Study in the Health Improvement Network. JAMA Dermatol. (2016) 152(3): 282-90. doi: 10.1001/jamadermatol.2015.4847

[35] Gelfand JM, Shin DB, Neimann AL, Wang X, Margolis DJ, and Troxel AB. The risk of lymphoma in patients with psoriasis. J Invest Dermatol. (2006) 126(10): 2194-201. doi: 10.1038/sj.jid.5700410

[36] Yang H, Brand JS, Li J, Ludvigsson JF, Ugalde-Morales E, Chiesa F, et al. Risk and predictors of psoriasis in patients with breast cancer: a Swedish population-based cohort study. BMC Med. (2017) 15(1): 154. doi: 10.1186/s12916-017-0915-4

[37] Fu Y, Lee CH, and Chi CC. Association of Psoriasis with Colorectal Cancer. J Am Acad Dermatol. (2020) S0190-9622(20): 32643-8. doi: 10.1016/j.jaad.2020.09.050

[38] Brauchli YB, Jick SS, Miret M, and Meier CR. Psoriasis and risk of incident cancer: an inception cohort study with a nested case-control analysis. J Invest Dermatol. (2009) 129(11): 2604-12. doi: 10.1038/jid.2009.113

[39] Khalid U, Ahlehoff O, Gislason GH, Skov L, Torp-Pedersen C, and Hansen PR. Increased risk of aortic valve stenosis in patients with psoriasis: a nationwide cohort study. Eur Heart J. (2015) 36(32): 2177-83. doi: 10.1093/eurheartj/ehv185

[40] Samarasekera EJ, Neilson JM, Warren RB, Parnham J, and Smith CH. Incidence of cardiovascular disease in individuals with psoriasis: a systematic review and meta-analysis. J Invest Dermatol. (2013) 133(10): 2340-6. doi: 10.1038/jid.2013.149

[41] Choi YM, Famenini S, and Wu JJ. Incidence of Pulmonary Arterial Hypertension in Patients with Psoriasis: A Retrospective Cohort Study. Perm J. (2017) 21: 16-073. doi: 10.7812/tpp/16-073

[42] Prodanovich S, Kirsner RS, Kravetz JD, Ma F, Martinez L, and Federman DG. Association of psoriasis with coronary artery, cerebrovascular, and peripheral vascular diseases and mortality. Arch Dermatol. (2009) 145(6): 700-3. doi: 10.1001/archdermatol.2009.94

[43] Honma M, Shibuya T, Iwasaki T, Iinuma S, Takahashi N, Kishibe M, et al. Prevalence of coronary artery calcification in Japanese patients with psoriasis: A close correlation with bilateral diagonal earlobe creases. J Dermatol. (2017) 44(10): 1122-8. doi: 10.1111/1346-8138.13895

[44] Ungprasert P, Srivali N, and Kittanamongkolchai W. Psoriasis and risk of incident atrial fibrillation: A systematic review and meta-analysis. Indian J Dermatol Venereol Leprol. (2016) 82(5): 489-97. doi: 10.4103/0378-6323.186480

[45] Armstrong AW, Harskamp CT, and Armstrong EJ. The association between psoriasis and hypertension: a systematic review and meta-analysis of observational studies. J Hypertens. (2013) 31(3): 433-42; discussion 442-3. doi: 10.1097/HJH.0b013e32835bcce1

[46] Khalid U, Ahlehoff O, Gislason GH, Kristensen SL, Skov L, Torp-Pedersen C, et al. Psoriasis and risk of heart failure: a nationwide cohort study. Eur J Heart Fail. (2014) 16(7): 743-8. doi: 10.1002/ejhf.113

[47] Shiba M, Kato T, Funasako M, Nakane E, Miyamoto S, Izumi T, et al. Association between Psoriasis Vulgaris and Coronary Heart Disease in a Hospital-Based Population in Japan. PLoS One. (2016) 11(2): e0149316. doi: 10.1371/journal.pone.0149316

[48] Kaiser H, Abdulla J, Henningsen KMA, Skov L, and Hansen PR. Coronary Artery Disease Assessed by Computed Tomography in Patients with Psoriasis: A Systematic Review and Meta-Analysis. Dermatology. (2019) 235(6): 478-87. doi: 10.1159/000502138

[49] Ungprasert P, Wijarnpreecha K, and Cheungpasitporn W. Patients with psoriasis have a higher risk of schizophrenia: A systematic review and meta-analysis of observational studies. J Postgrad Med. (2019) 65(3): 141-5. doi: 10.4103/jpgm.JPGM_253_18

[50] Cullen AE, Holmes S, Pollak TA, Blackman G, Joyce DW, Kempton MJ, et al. Associations Between Non-neurological Autoimmune Disorders and Psychosis: A Meta-analysis. Biol Psychiatry. (2019) 85(1): 35-48. doi: 10.1016/j.biopsych.2018.06.016

[51] Ungprasert P, Srivali N, and Kittanamongkolchai W. Risk of Parkinson's Disease Among Patients with Psoriasis: A Systematic Review and Meta-analysis. Indian J Dermatol. (2016) 61(2): 152-6. doi: 10.4103/0019-5154.177771

[52] Dowlatshahi EA, Wakkee M, Arends LR, and Nijsten T. The prevalence and odds of depressive symptoms and clinical depression in psoriasis patients: a systematic review and meta-analysis. J Invest Dermatol. (2014) 134(6): 1542-51. doi: 10.1038/jid.2013.508

[53] Hu SC, Chen GS, and Tu HP. Epidemiology of Depression in Patients with Psoriasis: A Nationwide Population-based Cross-sectional Study. Acta Derm Venereol. (2019) 99(6): 530-8. doi: 10.2340/00015555-3145

[54] Jensen P, Zachariae C, Skov L, and Zachariae R. Sleep disturbance in psoriasis: a case-controlled study. Br J Dermatol. (2018) 179(6): 1376-84. doi: 10.1111/bjd.16702

[55] Fleming P, Bai JW, Pratt M, Sibbald C, Lynde C, and Gulliver WP. The prevalence of anxiety in patients with psoriasis: a systematic review of observational studies and clinical trials. J Eur Acad Dermatol Venereol. (2017) 31(5): 798-807. doi: 10.1111/jdv.13891

[56] Alinaghi F, Tekin HG, Burisch J, Wu JJ, Thyssen JP, and Egeberg A. Global prevalence and bidirectional association between psoriasis and inflammatory bowel disease - A systematic review and meta-analysis. J Crohns Colitis. (2019) 14(3): 351-60. doi: 10.1093/ecco-jcc/jjz152

[57] Maybury CM, Porter HF, Kloczko E, Duckworth M, Cotton A, Thornberry K, et al. Prevalence of Advanced Liver Fibrosis in Patients With Severe Psoriasis. JAMA Dermatol. (2019) 155(9): 1028-32. doi: 10.1001/jamadermatol.2019.0721

[58] Yong WC, Upala S, and Sanguankeo A. Association between Psoriasis and Helicobacter pylori Infection: A Systematic Review and Meta-analysis. Indian J Dermatol. (2018) 63(3): 193-200. doi: 10.4103/ijd.IJD_531_17

[59] Ungprasert P, Wijarnpreecha K, and Kittanamongkolchai W. Psoriasis and Risk of Celiac Disease: A Systematic Review and Meta-analysis. Indian J Dermatol. (2017) 62(1): 41-46. doi: 10.4103/0019-5154.198031

[60] Acharya P, and Mathur M. Association between psoriasis and celiac disease: A systematic review and meta-analysis. J Am Acad Dermatol. (2020) 82(6): 1376-85. doi: 10.1016/j.jaad.2019.11.039

[61] Choudhary S, Pradhan D, Pandey A, Khan MK, Lall R, Ramesh V, et al. The association of metabolic syndrome and psoriasis: A systematic review and meta-analysis of observational study. Endocr Metab Immune Disord Drug Targets. (2019) 20(5): 703-17. doi: 10.2174/1871530319666191008170409

[62] Kiguradze T, Bruins FM, Guido N, Bhattacharya T, Rademaker A, Florek AG, et al. Evidence for the association of Hashimoto's thyroiditis with psoriasis: a cross-sectional retrospective study. Int J Dermatol. (2017) 56(5): 553-6. doi: 10.1111/ijd.13459

[63] Valduga JAG, Rebeiko LB, and Skare TL. Prevalence of Hashimoto's thyroiditis in psoriasis patients. Rev Assoc Med Bras (1992). (2021) 67(1): 52-7. doi: 10.1590/1806-9282.67.01.20200274

[64] Hu SC, Lin CL, and Tu HP. Association between psoriasis, psoriatic arthritis and gout: a nationwide population-based study. J Eur Acad Dermatol Venereol. (2019) 33(3): 560-7. doi: 10.1111/jdv.15290

[65] Mamizadeh M, Tardeh Z, and Azami M. The association between psoriasis and diabetes mellitus: A systematic review and meta-analysis. Diabetes Metab Syndr. (2019) 13(2): 1405-12. doi: 10.1016/j.dsx.2019.01.009

[66] Han JH, Lee JH, Han KD, Kim HN, Bang CH, Park YM, et al. Increased risk of psoriasis in subjects with abdominal obesity: A nationwide population-based study. J Dermatol. (2019) 46(8): 695-701. doi: 10.1111/1346-8138.14939

[67] Crincoli V, Di Comite M, Di Bisceglie MB, Fatone L, and Favia G. Temporomandibular Disorders in Psoriasis Patients with and without Psoriatic Arthritis: An Observational Study. Int J Med Sci. (2015) 12(4): 341-8. doi: 10.7150/ijms.11288

[68] Schell C, Schleich R, Walker F, Yazdi AS, Lerche H, Rocken M, et al. Restless legs syndrome in psoriasis: an unexpected comorbidity. Eur J Dermatol. (2015) 25(3): 255-60. doi: 10.1684/ejd.2015.2525

[69] Wu T, Duan X, Chen S, Chen X, Yu R, and Yu X. Association Between Psoriasis and Erectile Dysfunction: A Meta-Analysis. J Sex Med. (2018) 15(6): 839-47. doi: 10.1016/j.jsxm.2018.04.630

[70] Ungprasert P, Wijarnpreecha K, and Wetter DA. Periodontitis and risk of psoriasis: a systematic review and meta-analysis. J Eur Acad Dermatol Venereol. (2017) 31(5): 857-62. doi: 10.1111/jdv.14051

[71] Ogdie A, Harter L, Shin D, Baker J, Takeshita J, Choi HK, et al. The risk of fracture among patients with psoriatic arthritis and psoriasis: a population-based study. Ann Rheum Dis. (2017) 76(5): 882-5. doi: 10.1136/annrheumdis-2016-210441

[72] Keller JJ, Kang JH, and Lin HC. Association between osteoporosis and psoriasis: results from the Longitudinal Health Insurance Database in Taiwan. Osteoporos Int. (2013) 24(6): 1835-41. doi: 10.1007/s00198-012-2185-5

[73] Liu CY, Tung TH, Lee CY, Chang KH, Wang SH, and Chi CC. Association of Multiple Sclerosis with Psoriasis: A Systematic Review and Meta-Analysis of Observational Studies. Am J Clin Dermatol. (2019) 20(2): 201-8. doi: 10.1007/s40257-018-0399-9

[74] Grewal SK, Wan J, Denburg MR, Shin DB, Takeshita J, and Gelfand JM. The risk of IgA nephropathy and glomerular disease in patients with psoriasis: a population-based cohort study. Br J Dermatol. (2017) 176(5): 1366-9. doi: 10.1111/bjd.14961

[75] Ungprasert P, Srivali N, and Thongprayoon C. Association between psoriasis and chronic obstructive pulmonary disease: A systematic review and meta-analysis. J Dermatolog Treat. (2016) 27(4): 316-21. doi: 10.3109/09546634.2015.1107180

[76] Ungprasert P, and Raksasuk S. Psoriasis and risk of incident chronic kidney disease and end-stage renal disease: a systematic review and meta-analysis. Int Urol Nephrol. (2018) 50(7): 1277-83. doi: 10.1007/s11255-018-1868-z

[77] Shreberk-Hassidim R, Galili E, Hassidim A, Ramot Y, Merdler I, Baum S, et al. Epidemiology and Comorbidities of Psoriasis among Israeli Adolescents: A Large Cross-Sectional Study. Dermatology. (2019) 235(6): 488-94. doi: 10.1159/000501032

[78] Wang J, Ke R, Shi W, Yan X, Wang Q, Zhang Q, et al. Association between psoriasis and asthma risk: A meta-analysis. Allergy Asthma Proc. (2018) 39(2): 103-9. doi: 10.2500/aap.2018.39.4109

[79] Watad A, Bragazzi NL, McGonagle D, Damiani G, Comaneshter D, Cohen A, et al. Systemic Sclerosis is Linked to Psoriasis and May Impact on Patients' Survival: A Large Cohort Study. J Clin Med. (2019) 8(4): 521. doi: 10.3390/jcm8040521

[80] Darwazeh AM, Al-Aboosi MM, and Bedair AA. Prevalence of oral mucosal lesions in psoriatic patients: A controlled study. J Clin Exp Dent. (2012) 4(5): e286-91. doi: 10.4317/jced.50905
